# USP37 downregulation elevates the Chemical Sensitivity of Human Breast Cancer Cells to Adriamycin

**DOI:** 10.7150/ijms.54301

**Published:** 2021-01-01

**Authors:** Tao Qin, Xin-Ye Cui, Hao Xiu, Chao Huang, Zhen-Ni Sun, Xiao-Mei Xu, Lian-Hong Li, Lu Yue

**Affiliations:** 1 Department of Oncology, Qingdao Municipal Hospital, School of Medicine, Qingdao University, Qingdao, Shandong 266071, P.R. China.; 2 Department of General Surgery, The First Affiliated Hospital, Dalian Medical University, Dalian 116011, P.R. China.; 3 Department of Traditional Chinese Medicine, The West District of Qingdao Municipal Hospital Group (Qingdao Ninth People's Hospital), Qingdao, Shandong 266071, P.R. China.; 4 Department of Pathology, Dalian Medical University, Dalian 116044, P.R. China.; 5 The Key Laboratory of Tumor Stem Cell Research of Liaoning Province, Dalian Medical University, Dalian 116044, P.R. China.

**Keywords:** USP37, Adriamycin, Breast cancer, Bcl-2, Bax

## Abstract

**Background:** The evolution of adriamycin (ADR) resistance in the treatment of breast cancer often leads to a poor prognosis in patients. Ubiquitin-specific peptidase 37 (USP37) has been recently identified as a modulator in regulating the stemness of breast cancer cells, but its underlying mechanism remains unclear. In this study, we investigated whether USP37 knockdown could hamper the chemical resistance of MCF-7 and MCF-7/ADR cells to adriamycin and elucidated the potential mechanism.

**Methods:** Immunohistochemistry, western blotting, and RT-qPCR assays were performed to detect the USP37 expression in MCF-7 and MCF-7/ADR cells. The efficiency of USP37 knockdown in breast cancer cells was confirmed by western blotting and RT-qPCR assays. We also performed CCK-8 assay, flow cytometry, western blotting, and TUNEL assays to evaluate cell viability and apoptosis in breast cancer cells. *In vivo* study was performed to detect the tumorigenicity of MCF-7/ADR cells transfected with shScramble or shUSP37#1 under adriamycin treatment.

**Results:** Bioinformatic analysis indicated that USP37 overexpression was positively correlated with adriamycin resistance. The expression levels of USP37 in both MCF-7 and MCF-7/ADR cells increased significantly with the exposure to adriamycin in a dose-dependent manner. It was verified by the observation that USP37 downregulation elevated the inhibitory effects of adriamycin on breast cancer cells, suppressed cell proliferation caused by cell cycle arrest in G1/S transition, as well as induced apoptosis. Furthermore, *in vivo* study showed that knockdown of USP37 expression also decreased tumorigenicity of MCF-7/ADR cells in mice. TUNEL assay and observation of cell morphology magnified USP37 knockdown synergized with Adriamycin could elevate the apoptosis of MCF-7 and MCF-7/ADR cells. Western blotting assay illustrated that the combination of USP37 knockdown with adriamycin treatment significantly upregulated the expression levels of cleaved caspase 3 and Bax, whereas the expression level of Bcl-2 was inhibited.

**Conclusion:** Knockdown of USP37 gene expression can reverse the resistance of breast cancer cells to adriamycin, and down-regulating USP37 might be a valuable strategy against ADR resistance in breast cancer therapy.

## Introduction

Breast cancer is one of the most prevalent malignancies and is the second leading cause of cancer-related deaths, exerting severe threat on females' health 1. According to GlOBOCAM2018 statistics, 16% or 2.089 million newly diagnosed cancer patients in the globe were female with the diagnosis of breast cancer 2. According to the clinicopathological features of breast cancer, the therapies for breast cancer include tumor surgery, chemotherapy, radiotherapy, or some novel treatments are chosen as therapies for breast cancer. Chemotherapy is a paramount significance in the treatment of advanced breast cancer. Adriamycin, also known as hydroxydaunorubicin, is an anthracycline broad-spectrum chemotherapeutic drug 3, 4. Clinical trials have indicated that early breast cancer positively responds to both adriamycin monotherapy and its combined use with other chemotherapeutics. However, previous studies also showed that at least a quarter of breast cancer patients in the course of treatment developed adriamycin resistance, which has a negative effect on the prognosis of patients.

Breast cancer is a stem disease because certain tumor cells acquired stem-like properties and tumor-initiating proficiency 5. Collective evidence has indicated that therapeutic resistance, metastasis, and recurrence of breast cancer closely are associated with a subpopulation of cancer stem cells (CSCs) 6. Therefore, a better understanding of related stem makers and the molecular mechanism concerning the occurrence and progression of breast cancer will contribute to developing cancer therapeutics and improving the prognosis in patients diagnosed with breast carcinoma.

As a process of post-translational modification, protein deubiquitination plays an essential role in the biological activities, including cell differentiation, development and tumorigenesis. Ubiquitin-specific peptidase 37 (USP37), considered to be a deubiquitinating enzyme in the family of ubiquitin-specific proteases, contains α-helices with 20 amino acid sequences at its C-terminal. The α-helices bind to the ubiquitin protein and catalyze the hydrolysis of indicated proteins 7. Overexpressed USP37 can deubiquitinate and stabilize Cyclin A, thereby promoting the cell to transform from the G1 phase to the S phase 8. Recently, our group has found that the expression level of USP37 is exceptionally upregulated in CD44^+^/CD24^-^ phenotype breast cancer stem cells, and can regulate the stemness of breast cancer cell through its interaction with GLI-1^9^. It has been indicated that USP37 may be a new molecular marker in the gene-targeted therapy of breast cancer. However, it is not explicit whether the knockdown of USP37 gene expression can re-sensitize the adriamycin-resistant breast cancer cells.

In this study, the differential expression levels of USP37 gene in the adriamycin resistant cell line (MCF-7/ADR) and its corresponding cell line (MCF-7) were evaluated. We utilized a lentiviral system to knockdown USP37 gene in those cells. Cellular proliferation, cell cycle distribution, apoptosis and cell sensitivity to adriamycin* in vitro* were investigated. Furthermore, we examined the effect of USP37 downregulation on the tumorigenicity of MCF-7/ADR cells in BABL/c nude mice which received adriamycin treatment (5 mg/kg/week). Our data suggested that USP37 might be a therapeutic target agansist ADR resistance in breast cancer.

## Material and Methods

### Bioinformatic analysis

Gene transcripts were downloaded from The Cancer Genome Atlas (TCGA) for the breast cancer dataset. The database that contains the RNA-Seq gene-level data of 500 patients with invasive ductal breast carcinoma was utilized for Gene set enrichment analysis (GSEA). GSEA was carried out to predict the potential mechanism underlying the biological functions in positive- and negative-USP37 groups. The significant pathways enriched in each phenotype were selected based on the significant P value and normalized enrichment score (NES). The result pictures were downloaded from GSEA software of the Broad Institute 10.

### Cell Culture

Normal epithelial breast cell line MCF-10A and breast cancer cell line MCF-7 were purchased from the Cell Bank of Type Culture Collection of Chinese Academy of Sciences (Shanghai Institute of Cell Biology, Shanghai, China). MCF-10A cells were maintained in a mixed medium as per Qin's method 9. Adriamycin-resistant MCF-7/ADR cells were screened from MCF-7 cells in the optimal growth state after being exposed to different concentrations of adriamycin (MedChemExpress, Shanghai, China). MCF-7 cells were cultured in DMEM F12 supplemented with 10% FBS. MCF-7/ADR cells were cultured in RPMI 1640 media supplemented with 10% FBS. All media were supplemented with 100 unit/mL penicillin, and 100 μg/mL streptomycin, and all cells were incubated at 37 °C in a 5% CO_2_ atmosphere. Specifically, to sustain the multidrug resistant phenotype, MCF-7/ADR cells were cultured in the medium supplemented with adriamycin (4 μg/ml). Before *in vitro* assay, all cells were cultured in a drug-free medium for 24 hrs.

### Transient Transfection

In lentivirus transfection experiments, shRNAs used against USP37 included shRNA#1 (5'-CCG AAG AAC TGG AGT ATTC-3') and shRNA#2 (5'-CCT AGT AGT TCA CTA CAAT-3'). The cells were transfected with USP37 shRNAs or scramble shRNA (GenePharma Company, China) for 48 hrs before detection and analysis of targeted genes with RT-qPCR assay and western blotting assays.

### Proliferation Assay

Cells were seeded in a 96-well plate at a density of 1×10^3^ cells/well, and the cell viability was then assessed at different time points (0 hr, 24 hrs, 48 hrs, 72 hrs, 96 hrs and 120 hrs) by CCK-8 kit (Transgen, China). Each well was added with 10 μl of CCK-8 and 90 μl of fresh serum-free medium, followed by incubation for 4 hrs at room temperature. Afterward, we performed a Microplate Reader at 450 nm to measure the absorbance levels of samples.

### Cytotoxicity Assay

Breast cancer cells transfected with scramble shRNA or USP37#1 shRNA were seeded in a 96-well plate at a density of 5×10^3^ cells/well. With different concentrations of adriamycin pre-treatment for 24/48 hrs, the cells were cultured in a mixed liquid, containing 10 μl of CCK-8 and 90 μl of fresh serum-free medium. The absorbance levels of samples were measured by a Microplate Reader at 490 nm. Cell viability was calculated according to the manufacturer's instructions 9.

### Colony Formation Assay

Seventy-two hours after stable transfection, cells were grown in a six-well plate at a density of 1000 cells/well for two weeks. The culture medium was replaced every three days. Subsequently, the colonies were washed three times by the phosphate-buffered saline (PBS), fixed with methanol, and stained successively. The stained colonies were counted and photographed. The effect of USP37 on the colony formation was assessed by Student's t-tests in triplicate.

### Cell Cycle and Apoptosis Analysis by Flow Cytometry

MCF-7 cells were grown to 50% confluency and transfected with scramble shRNA or USP37#1 shRNA for 48 hrs. The harvested cells were then washed by cold PBS, centrifuged (2000rpm, 5min), and re-suspended at 1×10^6^/ml. Aliquot 1 ml cells were fixed with 70% ethanol for 2 hrs at 4 °C, washed by PBS and incubated with 50 μl of RNase A stock solution and 400 μl of propidium iodide staining solution (Key GEN Bio TECH, China) for 1 hour at 37 °C prior to the flow cytometry (FACS Calibur, BD Biosciences) analysis for cell cycle distribution. The apoptosis of MCF-7 and MCF-7/ADR cells was analyzed by the same flow cytometry after the washed cells were stained with FITC Annexin V and propidium iodide (Dojindo, Japan) per the manufacturer's instructions.

### Western Blotting Assay

The treated cells were washed with phosphate buffer saline to prepare for the cellular total protein extract. The samples scraped out of the culture flask were mixed with RIPA buffer (Beyotime, China) and cracked by an ultrasonic crusher for 10 mins on ice before protein concentration calculation with BSA kit (Transgen, China). A total 40 µg of protein sample was subjected to 10% SDS-PAGE, followed by transferring to polyvinylidene difluoride membranes (Millipore, Boston, MA, USA) at the end of electrophoresis. TBST (TBS and 0.01% Tween-2 0) dissolved with 5% non-fat milk was used to block the membranes for 60 mins at room temperature. Afterward, the blots were incubated overnight at 4°C with the primary antibodies (San Ying Biotechnology, China) diluted by USP37 (18465-1-AP) at 1:500, Bcl-2 (12789-1-AP) at 1:500, Bax (50599-2-Ig) at 1:500, caspase 3 (19677-1-AP) at 1:500 and β-tublin (10094-1-Ap) at 1:1000. Membranes were subsequently incubated with HRP-conjugated rabbit anti-IgG secondary antibodies (A23320, 1:16000) for 1 hr at 37 °C. Protein bands were detected using an enhanced chemiluminescence detection system (Amersham Pharmacia Biotech).

### RNA Extraction and Real-Time Quantitative PCR Analysis

The mRNA expression levels of USP37 and GAPDH were quantified by real-time quantitative PCR (RT-qPCR). The total RNA was extracted by Trizol reagent (Transgen, China) per the manufacturer's instructions. One μg of total RNA was reversely transcribed with All-in-one First-Strand cDNA Synthesis SuperMiX (Transgen, China). RT-qPCR was performed using the iCycler™ Real Time System (Bio-Rad Laboratories, Richmond, CA, USA) and a SYBR Premix EX Tag Master mixture kit (Transgen, China) according to the manufacturer's protocols. Primer pairs used for PCR amplification were USP37 F, 5'-GGC AGC AAG TCA TCA TTC CA-3' and USP37 R, 5'-GGC TGG TGA TGC AGG AAT TC-3'. GAPDH was used as an internal standard, with the primers GAPDH F, 5'-GGC ATC GTG ATG GAC TCC G-3'; GAPDH R, 5'-GCT GGA AGG TGG ACA GCG A-3'. The analysis of experimental results was carried out according to the 2^-ΔΔCt^ method 11.

### Experimental Animals

Six-week-old female BALB/c nude mice were purchased from Beijing Vital River Laboratory Animal Technology Co. Ltd. The mice were housed under specific pathogen-free conditions following the Guide for the Care and Use of Laboratory Animals (NIH). 1×10^7^ MCF-7/ADR cells were suspended with 0.1 ml serum-free media containing 50% Matrigel, and then injected subcutaneously into mammary fat pads. We divided the experiment into 2 groups, each of which contained 4 mice. To keep the consistency of experiment condition, adriamycin (5 mg/kg) was administered intraperitoneally (i.p.) once a week to mice for 21 days after inoculation and tumor formation. In the course of the experiment, one mouse died accidentally in the shScramble group. After 21 days of adriamycin administration, xenografted tumors were excised from sacrificed mice, weighed, and then stained with hematoxylin and eosin (H&E).

### TUNEL Assay

The apoptotic cells were detected by TUNEL BrightRed Apoptosis Detection kit (Transgen, China) following the manufacturer's instructions. Apoptotic cells were stained into red-positive cells. Cell nuclei were stained with 40, 6-diamino-2-phenylindole (DAPI, Sigma) staining. Representative images were collected.

### Immunohistochemistry Assay

For immunohistochemistry, the dish climbing glasses of cells were collected and fixed with 4% paraformaldehyde solution for 20 minutes at 4 °C. These samples were washed 3 times by PBS and then incubated with normal goat serum overnight. After being immersed in the diluted primary antibody for 2 h, they were incubated at 4 °C with the second antibody. The antibodies used in this experiment were anti-USP37 (1:500, 18465-1-AP) and goat anti-Rabbit IgG (H+L) (1:200; SA0000I-2) purchased from Proteintech Group Inc. Representative images were photographed and positive cells in the images were stained brown.

### Statistical Analysis

Statistical analysis was carried out by using GraphPad Prism 5.0 software (GraphPad Software, Inc., La Jolla, CA, USA). Each experiment was performed in triplicate. The data were statistically analyzed by Student's *t*-tests and expressed as mean ± SD. A *P*<0.05 was deemed to be statistically significant. *P<0.05, **P<0.01 and ***P<0.001 presented significant differences in the figures.

## Results

### Bioinformatic analysis of the USP37 related pathway

**Figure 1:**
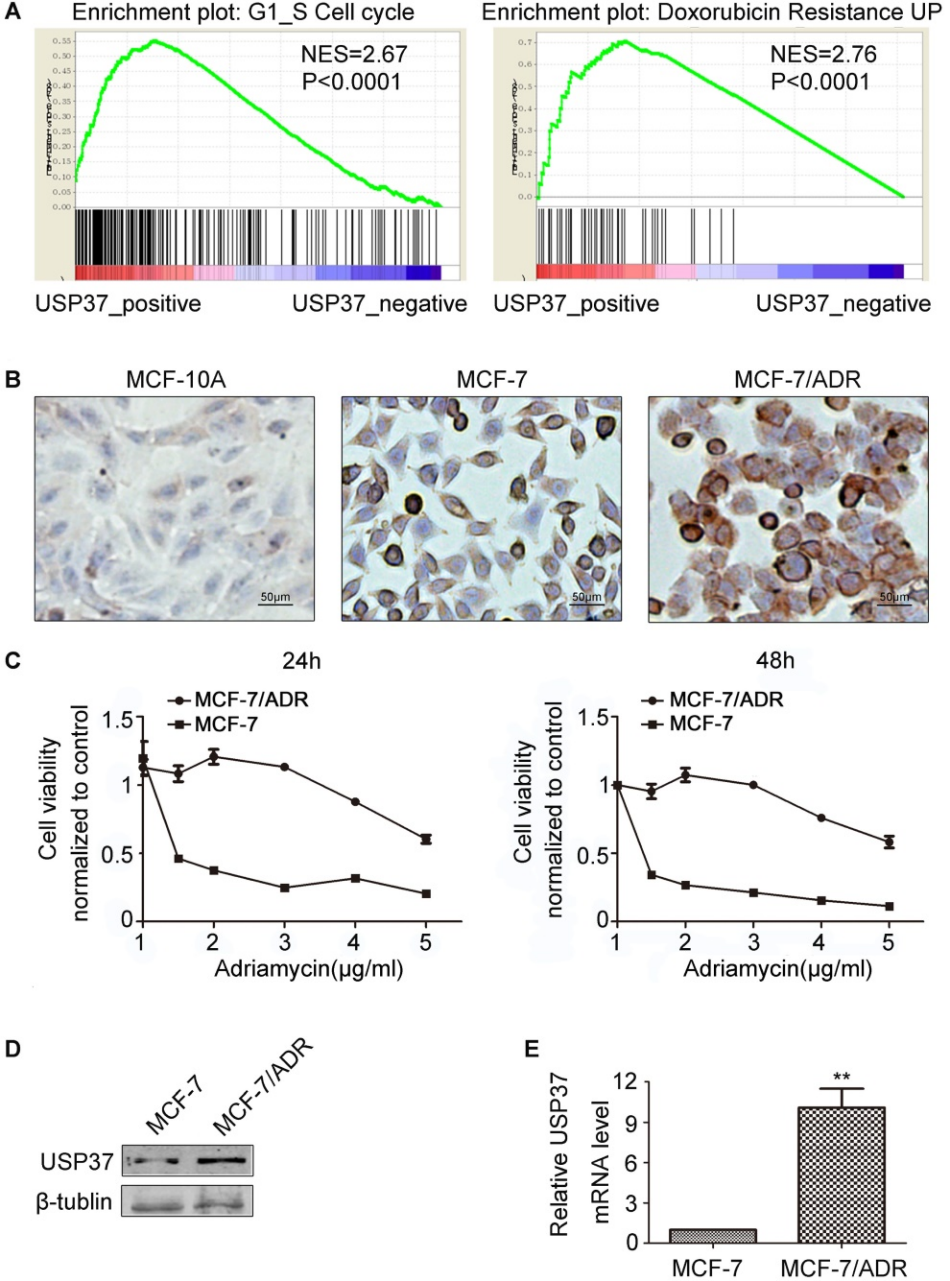
** Expression levels of USP37 gene in MCF-7 and MCF-7/ADR cells. (A)** The results of GSEA indicated that high USP37 expression strongly correlated with G1/S check point and adriamycin resistance. **(B)** Detection of USP37 gene expression in MCF10A, MCF-7 and MCF-7/ADR cells by immunohistochemistry. (Scale bars: 50 µm) **(C)** Detection of Viability of MCF-7 and MC7/ADR cells after adriamycin treatment for 24 hrs and 48 hrs by CCK-8 assay. **(D-E)** Detection of the mRNA and protein expressions of USP37 in MCF-7 and MCF-7/ADR cells by RT-qPCR and western blotting assay. **P<0.01 vs. corresponding MCF-7 cells.

USP37 expression in breast cancer samples was analyzed using the data collected from TCGA. After the collected data were divided into the USP37-positive group and the USP37-negative group, we identified the USP37 expression related mechanisms by applying GSEA. Analysis results indicated that USP37 overexpression was significantly correlated with G1/S checkpoint and adriamycin resistance (Figure 1A). The results achieved by bioinformatic analysis of invasive breast cancer data from TCGA suggested that knockdown of USP37 gene might interfere with cell cycle and chemoresistance to adriamycin.

### USP37 is highly expressed in the adriamycin resistant breast cancer cell line MCF-7/ADR

To confirm the relationship between USP37 gene and adriamycin resistance in breast cancer, USP37 expression levels were detected by western blotting and RT-qPCR assay. Firstly, we compared the chemical sensitivity of MCF-7 and MCF-7/ADR cells to adriamycin by CCK-8 assay. It was found that adriamycin remarkably inhibited the viability of these two cell lines in a dose-independent manner (Figure 1C). MCF-7/ADR cells with IC50 ranging from 3.783 μg/ml to 4.789 μg/ml were more resistant to adriamycin than MCF-7 cells with IC50 ranging from 0.1158 μg/ml to 0.2682 μg/ml. Secondly, the immunohistochemistry assay revealed that USP37 gene expression varied greatly among MCF-10A, MCF-7, and MCF-7/ADR cell lines. USP37 immunoreactivity was significantly higher in the cytoplasm of MCF-7/ADR cells than in the non-cancer MCF-10A cells (Figure 1B). Further results showed that the expression levels of USP37 in MCF-7/ADR cells were higher than that in MCF-7 cells (Figure 1D-E).

### USP37 knockdown strategy in MCF-7 and MCF-7/ADR cells

**Figure 2:**
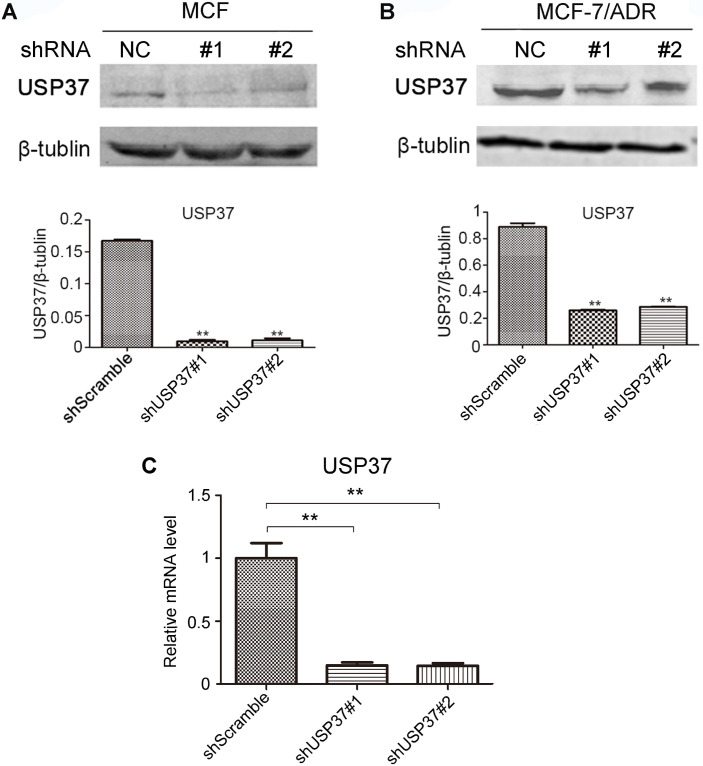
** Detection of the efficiency of USP37 knockdown in MCF-7 and MCF-7/ADR cells. (A-B)** Western blotting analysis showed USP37 knockdown efficiency in MCF-7 and MCF-7/ADR cells. **(C)** RT-qPCR analysis of USP37 knockdown efficiency in MCF-7 cells. **P<0.01 vs. shScramble group.

Some studies have demonstrated that USP37 is exceptionally overexpressed in some malignant tumor tissues, especially in lung cancer and breast cancer. To explore the cancerous role of USP37 gene in malignant tumor progression, USP37 gene was knocked down by a lentivirus system. The knockdown efficiency of USP37 gene is shown in Figure 2C. shUSP37#1 evidently inhibited the mRNA and protein levels of USP37 in MCF-7 cells and MCF-7/ADR cells (Figure 2A-B). Therefore, shUSP37#1 was used in follow-up tests.

### Knockdown of USP37 inhibits cell proliferation and induces apoptosis

**Figure 3:**
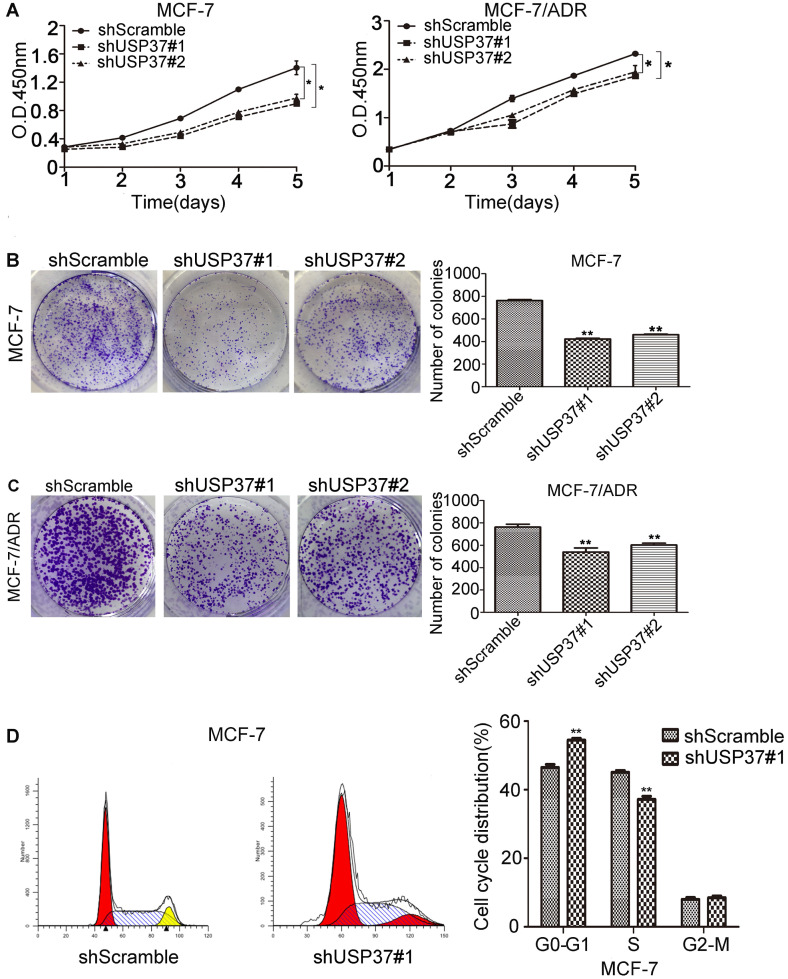
** USP37 knockdown reduced the proliferation of MCF-7 and MCF-7/ADR cells. (A)** Cells were transfected with shUSP37#1 and shUSP37. Cell proliferation was analyzed by CCK-8 assay. Cells transfected with shScramble were normalized to the control group. Data were presented as the mean±s.d. Student's *t*-test: *P<0.05 vs. shScramble group. **(B-C)** MCF-7 and MCF-7/ADR cells were transfected with USP37 shRNAs, and cell growth was measured by colony formation assay. The results were presented as the mean±s.d. Student's *t*-test: **P<0.01 vs. shScramble group. **(D)** USP37 downregulation led to arrest in G1/S phase of the cell cycle in MCF-7 cells. **P<0.01 vs. shScramble group.

**Figure 4:**
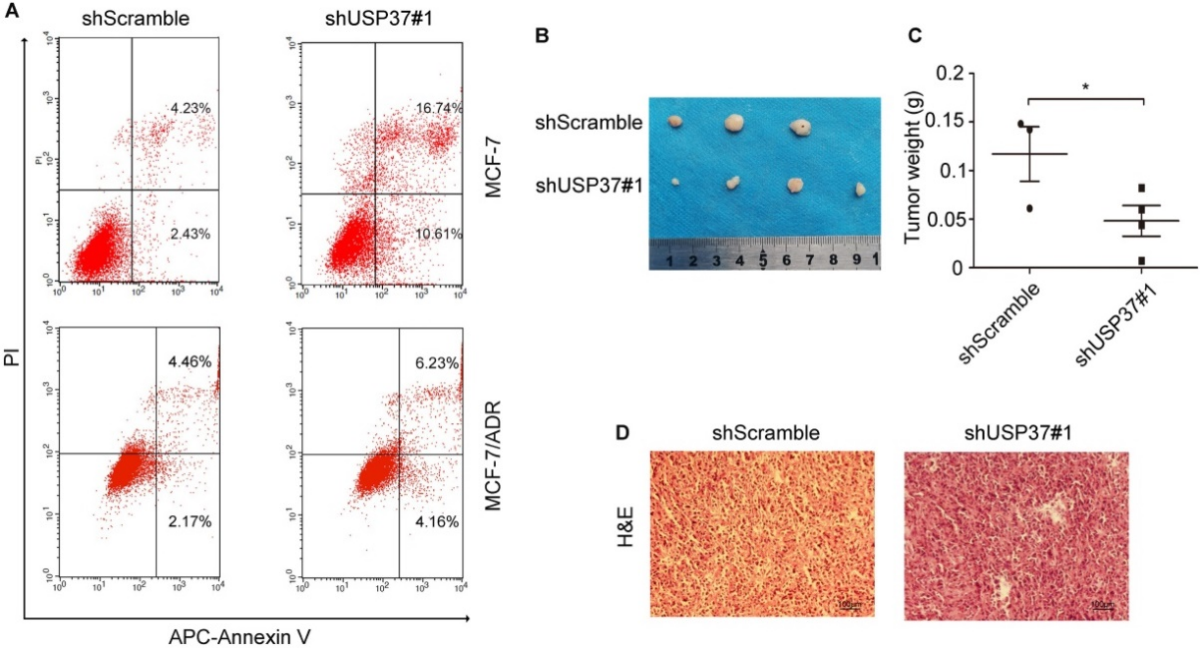
** Knockdown of USP37 induced cellular apoptosis and decreased tumor growth in nude mice. (A)** Flow cytometry showed that USP37 downregulation caused the apoptosis of MCF-7 and MCF-7/ADR cells. **(B-C)** Tumors were removed from the nude mice and taken photograph to show the sizes of xenografted tumors. Statistical analysis of the weight of tumor excised from BALB/c nude mice at day 21. *P<0.05 vs. shScramble group. **(D)** Images of HE staining in different groups. (Scale bars: 100 µm).

To investigate the latent role of USP37 gene in the transformation of breast cancer cells, cell proliferation was examined by CCK-8 assay. Knockdown of USP37 tremendously inhibited cellular proliferation as shown in Figure 3A. Besides, the anchorage-independent growth ability of breast cancer cells was detected by colony formation assay, which indicated that the number of colonies decreased following USP37 downregulation (Figure 3B-3C). Cell cycle disorders play an important role in the proliferation of malignant cells. To further verify the influence of USP37 downregulation on cell growth, MCF-7 cells infected with shScramble and shUSP37#1 were analyzed by flow cytometry analysis. The results suggested that knockdown of USP37 notably interfered with the G1/S phase transition in MCF-7 cells (Figure 3D). Furthermore, we detected cell apoptosis by flow cytometry analysis after USP37 was downregulated for 48 hrs, found that the early apoptosis ratio of the shUSP37#1 group (16.74%) was greatly higher than that of the shScramble group (4.23%) (Figure 4A). In MCF-7/ADR cells, the apoptosis rate of cancer cells with the shUSP37#1 group (6.23%) was also higher than that of the shScramble group (4.46%) (Figure 4A).

### USP37 knockdown inhibits the tumor formation of MCF-7/ADR cells *in vivo*

The above results indicated that USP37 gene was overexpressed in adriamycin-resistant breast cancer cells and its downregulation could inhibit cell growth. To confirm the tumor-inhibition effect of USP37 downregulation, MCF-7/ADR cells transfected with shUSP37#1 were injected into nude mice which received adriamycin treatment (3 mg/kg). After 21 days of observation, it was found that the size and the weight of xenografts in the shUSP37#1 group were significantly smaller than those in the shScramble group (Figure 4B-C). The data were in good agreement with the above experimental studies *in vitro.*

### Knockdown of USP37 reduces the chemoresistance of MCF-7 and MCF-7/ADR cells against adriamycin and activates the mechanism of apoptosis

**Figure 5:**
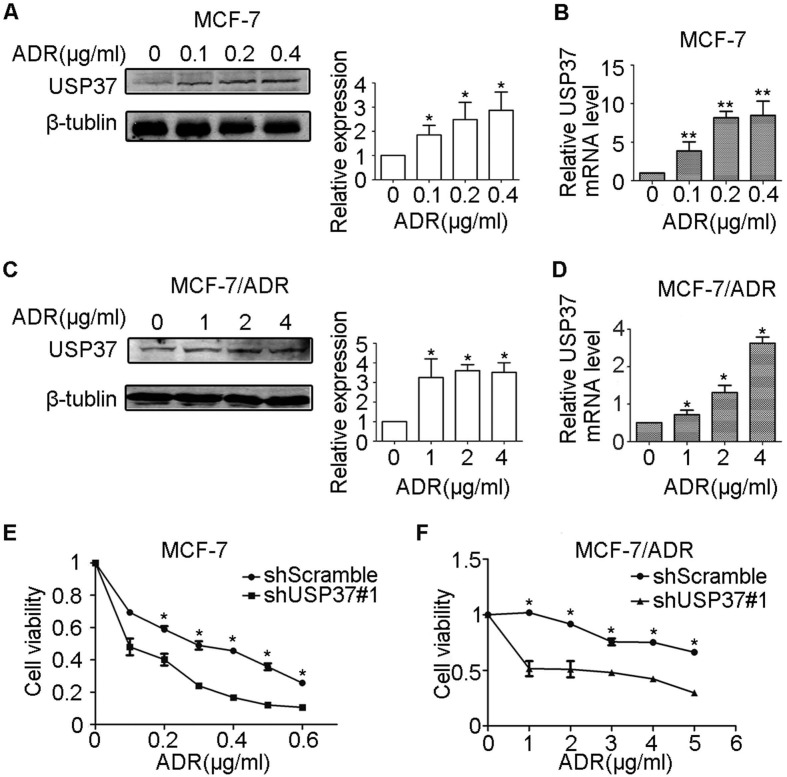
** The expression levels of USP37 were detected in MCF-7 and MCF-7/ADR cells treated with adriamycin, and its downregulation was related to chemical sensitivity. (A-D)** After exposure to adriamycin for 48 hrs, the mRNA and protein expression levels of USP37 were detected in MCF-7 **(A-B)** and MCF-7/ADR **(C-D)** cells. **(E-F)** Cells were stably transfected with shScramble and shUSP37#1 and then exposed to adriamycin of indicated doses for 48 hrs. Cell viability was measured by CCK-8 assay. **P<0.01, *P<0.05 vs. shScramble group.

**Figure 6:**
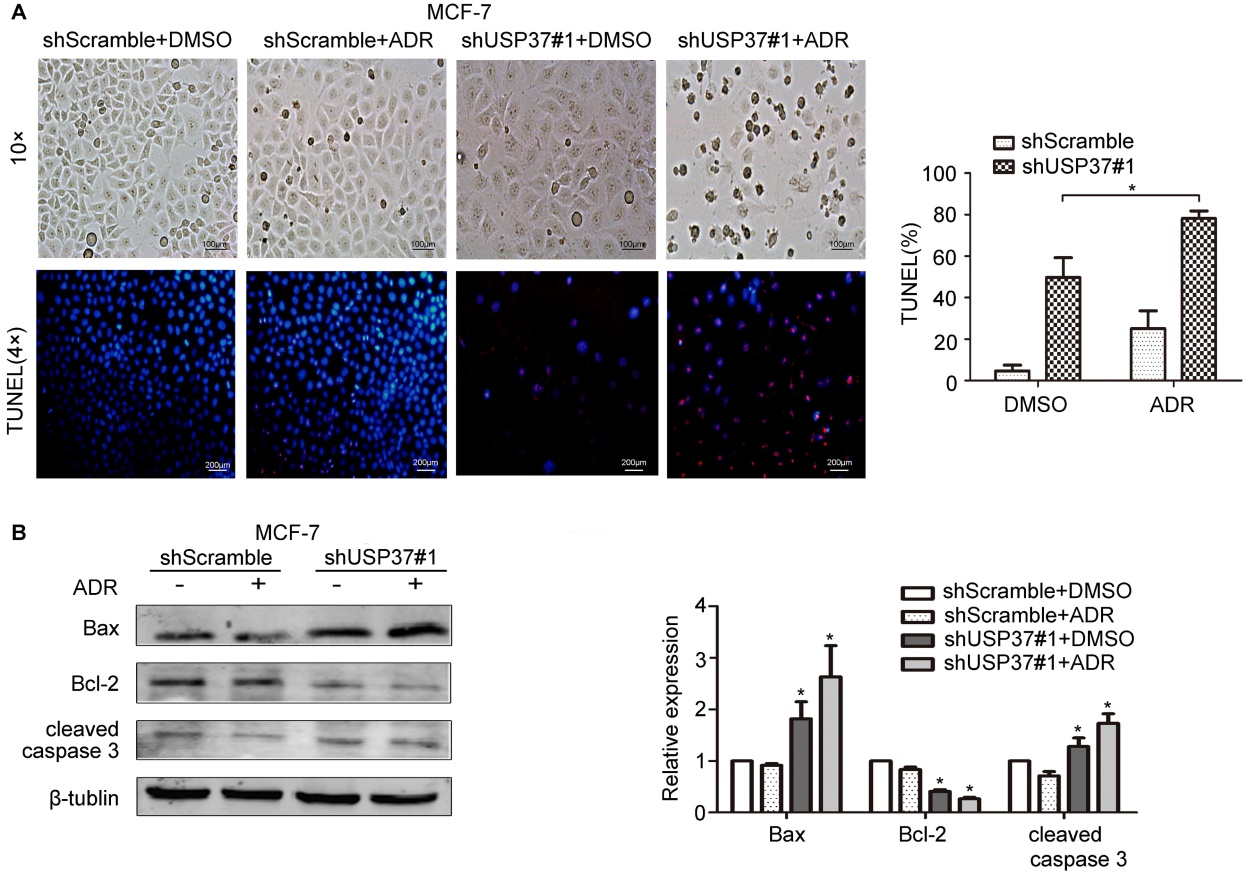
** Knockdown of USP37 reduced the chemoresistance of MCF-7 cells to adriamycin.** The transfected MCF-7 cells were pretreated with adriamycin (0.2 µg/ml). After the shScramble goup and the shUSP37#1 group were treated with dimethylsulfoxide (DMSO) or adriamycin for 48 hrs, **(A)** representative images of MCF-7 cells (Scale bars: 100 µm) and Tunel assay revealed the phenomenon of cellular apoptosis (Scale bars: 200 µm). Quantitative analysis results of TUNEL-positive signals are shown on the right. ^*^P<0.05 vs. shUSP37#1 group. **(B)** Western blotting assay was used to evaluate the protein expression levels of apoptosis-related genes (Bax, Bcl-2, cleaved caspase 3) and β-tublin. *P<0.05 vs. shScramble group.

**Figure 7:**
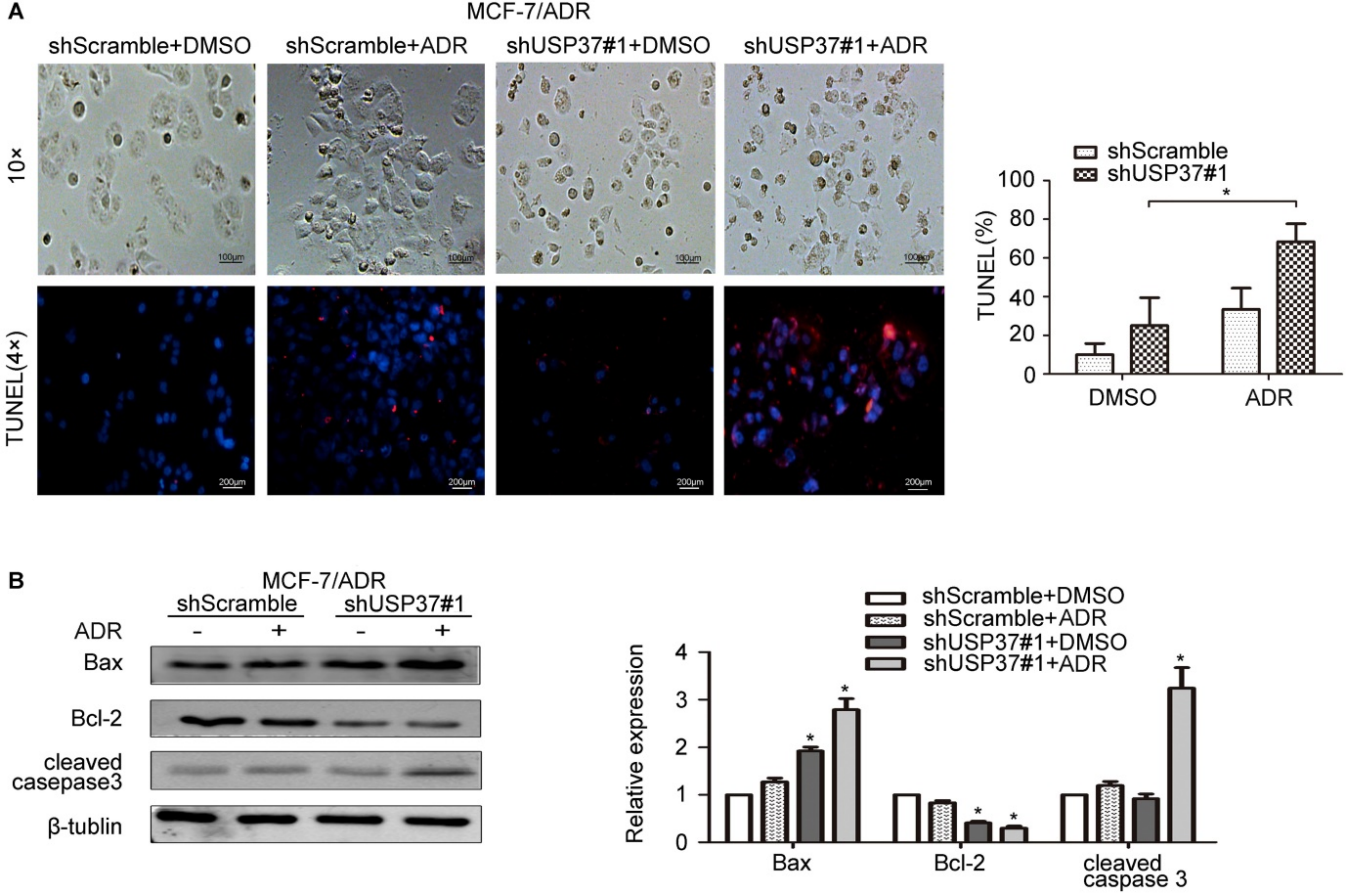
** Knockdown of USP37 reduces the chemoresistance of MCF-7/ADR cells to adriamycin.** The transfected MCF-7/ADR cells were pretreated with adriamycin (4 µg/ml). After the shScramble goup and the shUSP37#1 group were treated with dimethylsulfoxide (DMSO) or adriamycin for 48 hrs, **(A)** Representative images of MCF-7/ADR cells (Scale bars: 100 µm) and Tunel assay magnified the phenomenon of cellular apoptosis (Scale bars: 200 µm). Quantitative analysis results of TUNEL-positive signals are shown on the right. ^*^P<0.05 vs. shUSP37#1 group. **(B)** Western blotting assay was used to evaluate the protein expression levels of apoptosis-related genes (Bax, Bcl-2, cleaved caspase 3) and β-tublin. *P<0.05 vs. shScramble group.

USP37 gene expression in both MCF-7 and MCF-7/ADR cells was markedly upregulated by the exposure to adriamycin in a dose-dependent manner (Figure 5A-D). The above data revealed that MCF-7/ADR cells with USP37 overexpression were less sensitive to adriamycin than MCF-7 cells with low USP37 expression. To further detect whether knockdown of USP37 could regulate the chemical sensitivity of breast cancer cells to adriamycin, USP37 gene was downregulated by a lentivirus system. We observed that MCF-7 cells with USP37 knocked down (IC50: 0.1064 μg/ml) were more sensitive to adriamycin compared to MCF-7 cells transfected with shScramble (IC50: 0.2718 μg/ml). Furthermore, IC50 in MCF-7/ADR cells with downregulated USP37 (0.9229-2.839 μg/ml) was evidently lower than that of the shScramble group (5.662-9.153 μg/ml) (Figure 5E-F). After the shUSP37#1 group was treated with adriamycin or DMSO for 48 hrs, the morphologies of MCF-7 and MCF-7/ADR cells in the combination group (shUSP37#1+ adriamycin treatment) were seriously damaged compared with the other three groups (Figure 6A and Figure 7A).

Tunnel assay also confirmed that the population of apoptotic MCF-7 and MCF-7/ADR cells treated with shUSP37#1+adriamycin was higher than that of the shUSP37#1 group (Figure 6A and Figure 7A). Further investigation showed that shUSP37#1 can activate the expression levels of cleaved caspase 3 and Bax, and inhibit Bcl-2 expression (Figure 6B, 7B). Consistent with the above results, it was observed a synergistic effect on increase of Bax expression and reduction of Bcl-2 expression in the group receiving both shUSP37#1 and adriamycin treatment. These results demonstrated that USP37 knockdown could reduce the chemoresistance of breast cancer cells against adriamycin.

## Discussion

Chemoresistance not only leads to disease recurrence and metastasis but also undermines the expected therapeutic effects of chemotherapeutic agents 12. However, it is seemingly difficult to overcome in cancer therapies. Chemoresistance is generally classified into two types, namely, primary resistance and acquired resistance, which are attributed to intratumoral heterogeneity, including oncogenes, mitochondrial alteration, epithelial-mesenchymal transition (EMT), and the presence of CSCs 13. Studies have indicated that conventional treatments enhance the CSCs characteristics of non-CSCs and promote the conventional non-CSCs to transform into CSCs 14, 15. Preliminary studies of our team have established that USP37 regulates the stemness, cell invasion, and EMT in breast cancer cells 9. In this study, we observed that the expression levels of USP37 were increased in MCF-7 and MCF-7/ADR cells treated with adriamycin in a dose-dependent manner. Moreover, it was also implicated that USP37 knockdown was capable of inducing intrinsic apoptosis in MCF-7 and MCF-7/ADR cells. Further experiment results showed that the downregulation of USP37 increased the sensitivity of MCF-7/ADR cells to adriamycin *in vitro* and inhibited the tumor growth of MCF-7/ADR cells *in vivo*. Therefore, we concluded that USP37 gene plays a critical role in ADR resistance of breast cancer.

Recently, a series of studies have demonstrated that USP37 is involved in carcinoma occurrence and progression 16. In a study about the pathogenesis of lung cancer, highly-expressed USP37 was proven to promote cell proliferation, enhance the Warburg effect, and increase the mortality rate and metastasis 17. Through knockdown of USP37 expression in breast cancer MCF-7 cells and MCF-7/ADR cells with a lentivirus vector system, we demonstrated that USP37 downregulation can evidently inhibit cellular proliferation of breast cancer cells. By *in vivo* assay, USP37 downregulation also suppressed the tumor growth of MCF-7/ADR cells. The results are consistent with the previous reports that USP37 knockdown significantly inhibited the tumor formation of malignancies, such as lung cancer 18, kidney cancer 19, hepatocellular cancer 20. Inordinate cell cycle progression resulted in tumorigenesis 21. The current finding showed that USP37 downregulation leads to disorder of cell cycle during G1 and G1/S phases of the cell cycle 22, which confirmed our data that USP37 knockdown altered the cell cycle by increasing the accumulation of the G0/G1 phase and reducing the cell population in S phase.

Conventionally, USP37 gene was regarded as a stem marker and its overexpression maintained the stemness of breast cancer 9. The present study focused on the role of USP37 in adriamycin resistance of breast cancer. It was noteworthy that we observed a positive correlation between USP37 overexpression and adriamycin resistance by bioinformatic analysis. MCF-10A cells are normal breast epithelium cells, characterized by tumorigenicity deficiency in nude mice and sensitivity to the toxic effects of chemical drugs 23. We found that USP37 immunoreactivity was the lowest in MCF-10A cells among the three types of breast cancer cells studied in this paper. Additionally, the expression levels of USP37 in MCF-7/ADR cells were higher than those in MCF-7 cells. USP37 gene expression changed greatly with the increase of adriamycin concentration. Further experiments confirmed that USP37 downregulation reduced the chemo-resistance of MCF-7/ADR cells to adriamycin. These data raised a question whether USP37 overexpression induced by adriamycin treatment could interfere with adriamycin-induced cell apoptosis.

Since the dysregulation of apoptosis also leads to cancer cell growth, anti-apoptosis is the underlying reason for tumor resistance to therapies. The tumor suppressor gene P53 tends to induce cellular apoptosis in response to cellular stress 24, so the loss of P53 functional gives rise to the anti-apoptosis response and subsequently cancer therapy resistance. P53 is deemed to play a critical role in the apoptotic function of the endogenous pathway because it regulates pro-survival and pro-apoptotic Bcl-2 family members, including Bax 25. Besides, the adriamycin resistance of breast cancer cells is related to upregulated Bcl-2 and downregulated Bax 26. Our data demonstrated that USP37 downregulation enhanced the inhibitory effect of adriamycin on MCF-7 and MCF-7/ADR cancer cells through the inhibition of Bcl-2/Bax signaling pathway. Cleaved caspase 3 is involved in activating the Caspase cascade of the apoptotic pathway 27. Our results elucidated that USP37 downregulation combined with adriamycin obviously destructed cancer cell morphology, and enhanced the expression of cleaved caspase 3. It was suggested that knockdown of USP37 expression combined with adriamycin treatment was closely correlated with adriamycin sensitivity of breast cancer. Taken together, USP37 upregulation was involved in adriamycin resistance of breast cancer cells, and USP37 might be a prospective therapeutic biological marker for monitoring and overcoming breast cancer adriamycin resistance.

## Conclusions

In this study, we found USP37 is over-expressed in adriamycin-resistant breast cancer cells and the increase showed a dose-dependent manner with the exposure to adriamycin. More significantly, knockdown of USP37 will impair the adriamycin resistance of breast cancer cells and induce intrinsic apoptosis, including Bcl-2/Bax/cleaved caspase 3. These findings suggest that USP37 may act as a potential gene target against chemoresistance in breast cancer therapies.

## Abbreviations

USP37: ubiquitin-specific peptidase 37
CSCs: cancer stem cells
GLI-1: glioma-associated oncogene homolog 1
GSEA: gene set enrichment analysis
DMSO: dimethylsulfoxide
ADR: adriamycin
EMT: epithelial-mesenchymal transition
BC: breast cancer
